# Effect of the 3rd Dimension within the Representative Volume Element (RVE) on Damage Initiation and Propagation during Full-Phase Numerical Simulations of Single and Multi-Phase Steels

**DOI:** 10.3390/ma14010042

**Published:** 2020-12-24

**Authors:** Faisal Qayyum, Muhammad Umar, Sergey Guk, Matthias Schmidtchen, Rudolf Kawalla, Ulrich Prahl

**Affiliations:** 1 Institute of Metal Forming, Technische Universität Bergakademie Freiberg, 09599 Freiberg, Germany; sergey.guk@imf.tu-freiberg.de (S.G.); Matthias.Schmidtchen@imf.tu-freiberg.de (M.S.); Rudolf.Kawalla@imf.tu-freiberg.de (R.K.); Ulrich.Prahl@imf.tu-freiberg.de (U.P.); 2 Department of Mechanical Engineering, Khwaja Fareed University of Engineering and Information Technology, Rahim Yar Khan, Punjab 64200, Pakistan; muhammad.umar@kfueit.edu.pk

**Keywords:** crystal-plasticity, DAMASK, representative volume element, dual-phase steel, damage behaviour, modeling, local distribution, stress, strain, statistical data analysis

## Abstract

In this research, the effect of 2D and 3D Representative Volume Element (RVE) on the ductile damage behavior in single-phase (only ferrite) and dual-phase (ferrite and martensite) steels is analyzed. Physical and fitting parameters of the constitutive model for bcc-ferrite and bcc-martensite phases are adapted from the already published work. Crystal plasticity (CP) based numerical simulations without damage consideration are run and, later, ductile damage criteria for the ferrite phase is defined for all cases. The results of the non-damage (-nD-) and damage (-D-) simulations are compared to analyze the global and local differences of evolving stresses and strains. It is observed that for the same model parameters defined in all cases, damage initiation occurs at the overall higher global strain in the case of 3D compared to 2D. Based on statistical data analysis, a systematic comparison of local results is carried out to conclude that the 3D RVEs provide better quantitative and qualitative results and should be considered for such full phase simulations. Whereas 2D RVEs are simple to analyze and provide appropriate qualitative information about the damage initiation sites.

## 1. Introduction

Steel is extensively used as a structural material due to wide availability, low cost, and high strength [1]. The varying microstructure of steel results in varying mechanical properties [2,3]. Such varying microstructure also yields different damage mechanisms, crucial for many components operating at critical loading conditions, especially in the forming industry [4,5]. The development of application-focused advanced steels requires an accurate understanding of the microstructure-property relationship and resulting damage behavior in emerging modern steels.

Generally, single-phase steels are soft and ductile. Plastic deformation in such materials occurs due to dislocation motion or twin formation [6]. The damage initiation in such materials has been reported to start from the high angle grain boundaries [6]. The damage evolution occurs due to the void coalescence in close proximity, forming micro-cracks. These cracks act as a stress localization zone, and under applied incremental load, the material fails due to rupture [7,8]. Ductile damage in such materials can be identified by the typical cup and cone-shaped marks on the fracture surface [9,10]. Single-phase steels are not very engineering-friendly materials due to their low strength and corrosion resistance [3]. Still, they can be used as an example to analyze and understand the material deformation and damage behavior in single-phase materials.

DP-steels are preferred in most engineering applications due to their high strength and appropriate ductility [3], which are desired by the load-carrying components in severe working conditions. In such materials, islands of very high strength martensite are present, which pin the moving dislocations during plastic deformation. The pinning of dislocations on the martensite/ferrite interfaces results in high strain hardening [11]. The damage in such materials has been reported to initiate due to multiple phenomena taking place at the same time [4,12] i.e., (a) ductile damage of the ferrite matrix, (b) decohesion of the martensite/ferrite interface, and (c) brittle cracking of the martensite under hydrostatic stress conditions. Stress accumulation on the tips of the initiated cracks further accelerates the material failure, and eventual rupture of the component occurs under externally applied loading conditions. This kind of ductile-brittle damage evolution has been reported by several researchers after the failure analysis of DP-steels [7,8]. These deformation, damage, and failure mechanisms are majorly microstructure dependent [13,14]. It has been reported that changing the carbon content in the martensite phase, alloy composition, processing route, microstructure, and orientation distribution results in entirely different mechanical properties during deformation.

Invaluable research has already been carried out to identify the microstructure-property relationship in the crystalline materials, especially steels [15]. Generally, the research route follows manufacturing a particular material, followed by the attribute and property-based microstructural and mechanical analysis. This generally followed path is a uni-directional method with its limitations, such as higher experimentation costs and comparatively fewer output data. Crystal plasticity (CP) based advanced numerical simulation tools with recently developed methods have empowered the research community to carry out this task successfully for certain material classes [16,17,18,19,20]. However, there is still a long way to go. There are challenges associated with this method, as well. The major are: (a) identification of the CP model parameters for each phase, and (b) selection of an appropriate representative volume element (RVE), which should be small enough for reduced computation costs and large enough to be inclusive of all the essential material microstructure features. In a few recent publications [21,22,23] the authors tried to address this issue and identified appropriate RVEs for single and multi-phase materials.

Full phase simulations for the single and multi-phase steels have been carried out earlier by other researchers. They demonstrated that such models could be used for analyzing material deformation behavior to a specific deformation degree [17,24,25]. A little work has been carried out with the damage criteria definition for single and multi-phase materials. Mostly for the 2D case [26,27,28] because it is easier to measure, visualize, and compare. Characterization of local strains and especially damage initiation and evolution in 3D is a tedious task. A systematic study for such a case is still missing to the best of the author’s knowledge.

Incorporating damage in CP simulations by implementing an appropriate mathematical model with acceptable accuracy is a challenging task. Several researchers in the recent past has contributed their valuable research results to address these challenges [29,30]. The optimal RVE size is crucial, and therefore in continuation of the previous publication [23], this study is being carried out in a systematic order to incorporate damage criteria on the previously identified optimal RVEs.

In this work, the effect of 2D and 3D RVEs on the ductile damage behavior in single and multi-phase steels are studied and presented. The ductile damage criterion for the ferrite phase in both steel cases is defined. A systematic comparison of local results to identify damage initiation zones and damage propagation behavior is carried out. The details of material data, RVE construction, simulation scheme, and CP material model parameters are provided in Section 2. Section 3 presents the results obtained in this study. In Section 4, the results are discussed in comparison with the state of the art method, and an insight into the outlook is provided. Eventually, the study is concluded in Section 5.

## 2. Numerical Simulation Model Development

Ferrite-steel as a single-phase and DP-steel as multi-phase are chosen as case study materials in the current work. They were chosen to analyze the effect of RVE thickness and damage criteria selection on single and multi-phase materials. For the numerical simulation modeling, RVEs are virtually constructed using the open-source tool Dream.3D 6.5.121 [31]. The constructed RVEs are sliced into geometries with 01-layer and 50-layers thicknesses according to the methodology published earlier [23]. These RVEs are used as input geometries for the numerical simulation models implemented separately in both cases. Crystal plasticity (CP) framework based open-source numerical simulation tool (DAMASK) is used to carry out full phase simulations according to the already published methodology [32]. For readers not familiar with the modeling strategy, a brief background of the model is provided in the Appendix A. Micro-structural attributes for ferrite and martensite phases needed during CP based simulations are adopted from literature [33]. Damage parameters for the ferrite phase are adopted from the previous work of Shanthraj et al. [30]. The study results are critically analyzed by comparing the global and local stress–strain and damage evolution in all cases.

### 2.1. RVE Construction and Geometry Files Production

The values for microstructural parameters, i.e., grain size and martensite percentage, were adopted from the previous work of Jiang et al. [33]. Dream.3D was used to construct virtual RVEs with a ferrite grain size of average estimated sphere diameter $D_{f}$ 10.7 μm. A pipeline was built with the initialization of virtual data generation using a stats generator filter. For steel, only the ferrite phase was generated, whereas, for the DP-steel case, the ratio of 90 vol.% primary phase (ferrite) and 10 vol.% secondary phase (martensite) was selected. Both phases were generated as cubic equiaxed crystal structures, and ellipsoid grain shape types were selected, while the maximum number of iterations (swaps) allowed was 100,000. Contrary to other similar tools [34], the Dream.3D algorithm automatically caters for the homogeneous distribution of martensite phase in the ferrite matrix and avoids clustering of second phase particles. Using already published methodology [35], the generated RVEs were recorded as .xdmf file, which is readable by Paraview for visualization, and .geom files were saved, which are readable by DAMASK.

**Figure 1:**
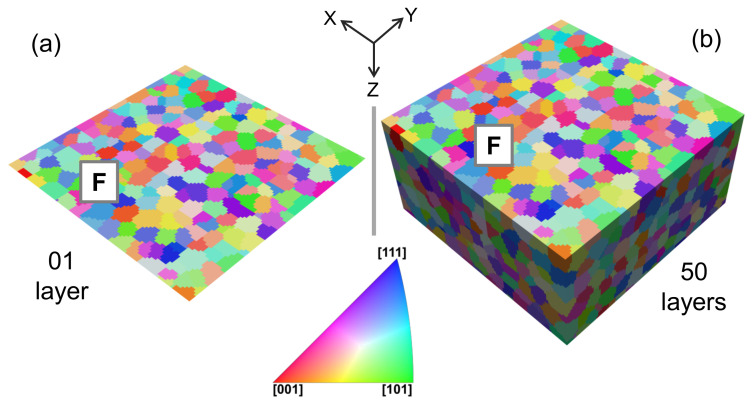
Steel RVEs in inverse pole figure (IPF) colors (**a**) 2D RVE with 1 μm thickness (**b**) 3D RVE with 50μm thickness.

**Figure 2:**
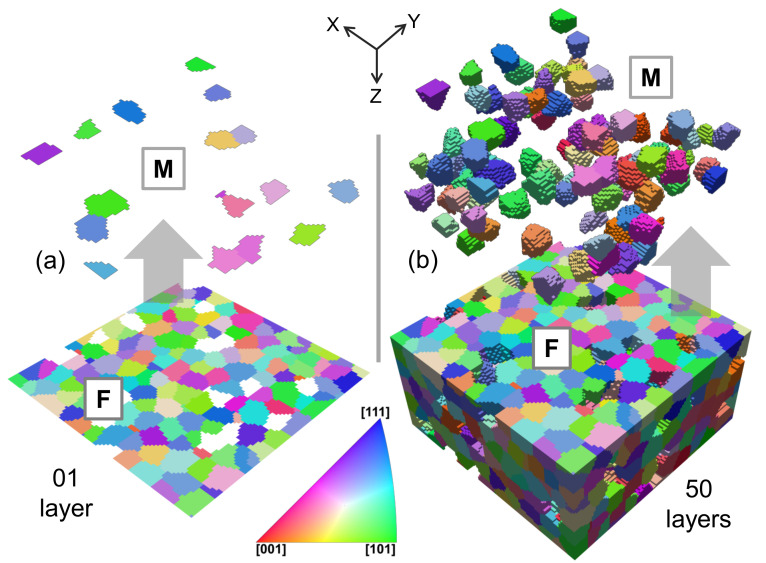
DP-steel RVEs in IPF colors. Both phases are shown separately for better visualization of the phase distribution in the generated RVEs, where (F) represents ferrite phase and (M) represents the martensite phase. Here, (**a**) 2D RVE with 1μm thickness (**b**) 3D RVE with 50 μm thickness.

The constructed RVEs for steel are shown in Figure 1 and the constructed RVEs for the DP-steel are shown in Figure 2. The total number of grains in the 2D RVEs are ≈150, and in 3D RVEs are ≈1500. The mesh density in all the generated RVEs is $1\frac{element}{m^{3}}$. In the previous study by Qayyum et al. [23] it has already been established that the 3D RVEs with 100×100×50 size comprising of ≥1000 grains behave isotropically when loaded in any direction. Due to an overall neutral orientation distribution function (ODF) selection during RVE generation, a complete random orientation distribution of the grains is observed. Martensite and ferrite phases in Figure 2 are shown separately for better visualization of their distribution in the RVEs.

### 2.2. Material Properties

Although there is a large difference in the mechanical properties of ferrite and martensite phases, in current research, both phases were assigned elastic-viscoplastic properties by adopting already developed and validated phenomenological power-law based material models available within the framework of DAMASK [20]. The elastic coefficients, variables defining plastic flow behavior, and fitting parameters were adopted from the already published literature [32]. The adopted parameters are presented in Table 1. To incorporate damage, the already developed model by Roters et al. [16] was adopted and incorporated in the material files of respective RVEs. The adopted parameters are presented in Table 2. This isotropic ductile damage model is based on the total accumulated plastic slip at a material point, where the local damage $\phi_{l}$ is given by:

(1) $$\phi_{l}=min\left(1,\frac{\epsilon_{crit}}{\Sigma_{a=1}^{nss}\gamma^{\alpha }}\right)$$

The value of critical plastic strain $\epsilon_{crit}$ for the ferrite phase was adopted from the published work of Tasan et al. [32] for the current case is stated to be 0.5. The damage phase field value $\phi_{l}=0$ corresponds to fully degraded material stiffness, whereas $\phi_{l}=1$ refers to the fully coherent bulk material.

### 2.3. Loading Conditions, Processing, and Post Processing

In the current work, plane stress mixed boundary conditions where applied with tensile loading along x-direction while keeping the out-of-plain surfaces in all geometries stress-free. The periodic boundary conditions were stated as:

(2) $${\dot{F}}_{ij}=\left[\begin{array}{ccc} 1 & 0 & 0\\ 0 & * & 0\\ 0 & 0 & * \end{array}\right]\times 10^{-3}\;\cdot \;s^{-1}$$

(3) $$P_{ij}=\left[\begin{array}{ccc} {}* & * & *\\ {}* & 0 & *\\ {}* & * & 0 \end{array}\right]Pa$$

where, ${\dot{F}}_{ij}$ and $P_{ij}$ are the macroscopic rate of the deformation gradient and macroscopic first Piola–Kirchhoff stress tensor, respectively. The coefficients denoted by ‘*’ highlight the stated complimentary conditions. Using these conditions, uni-axial tensile load was applied in x-direction, with $1\times 10^{-3}$ s^−1^ iso-static strain rate.

For simulations without damage, fewer homogeneous number of increments were recorded to construct the flow curves without damage consideration. The simulations with damage comparatively higher number of increments were recorded after the damage initiation in a given RVE. It was done to record the material degradation phenomenon in detail. This made the damage simulations slow and highly computing-intensive, which crashed after specific material degradation had occurred in the RVE. The results in this work are presented up to the point for which the simulation results converged. The readers are encouraged to refer to the earlier work [17] for the explanation of the assumptions in the fast Fourier transformation (FFT) regarding the reference stiffness used within the framework of the crystal plasticity provided by DAMASK.

The results were post-processed using already available subroutines in the DAMASK installation module, and data were further statistically analyzed using the Seaborn library in Python. The local stress–strain distribution plots were constructed using the open-source tool Paraview [37].

## 3. Results

In the current research, simulations on single-phase steel and multi-phase DP-steel were run for the case of 2D and 3D RVEs with and without damage criteria incorporation. Two types of comparisons were made in this research:

1. Damage initiation and propagation behavior in single-phase steel compared with non-damage simulation results. The results were recorded to see how do the 2D and 3D RVE considerations—in the case of single-phase steel—affect the damage evolution, and,
2. Similarly, for the case of DP-steel, 2D, and 3D RVEs with damage and non-damage criteria are simulated to observe the difference in damage initiation and propagation for the case of multi-phase materials.

Considering the recently published study of Qayyum at al. [23], consideration of 3D RVEs for accurate analysis and more realistic local stress and strain evaluation is necessary. Therefore, a systematic study comparing damage behavior in 2D and 3D RVEs for checking the damage initiation sites, damage propagation rates, merits, and demerits for choosing a specific RVE is needed. The current work focuses on structurally developing such analysis using statistical data processing of simulation results.

To systematically analyze and compare the results of such an extensive study, a structured nomenclature is developed as provided in Table 3, which simplifies the reference, comparison, and understanding of the presented results. In the following sections, results for the cases of single-phase steel and DP-steel are discussed separately. In the discussion section of the article, the similarities and differences in the trends are covered by comparing them with already reported results.

### 3.1. Deformation and Damage Behavior of Single-Phase Steel

#### 3.1.1. Global Behavior

**Figure 3:**
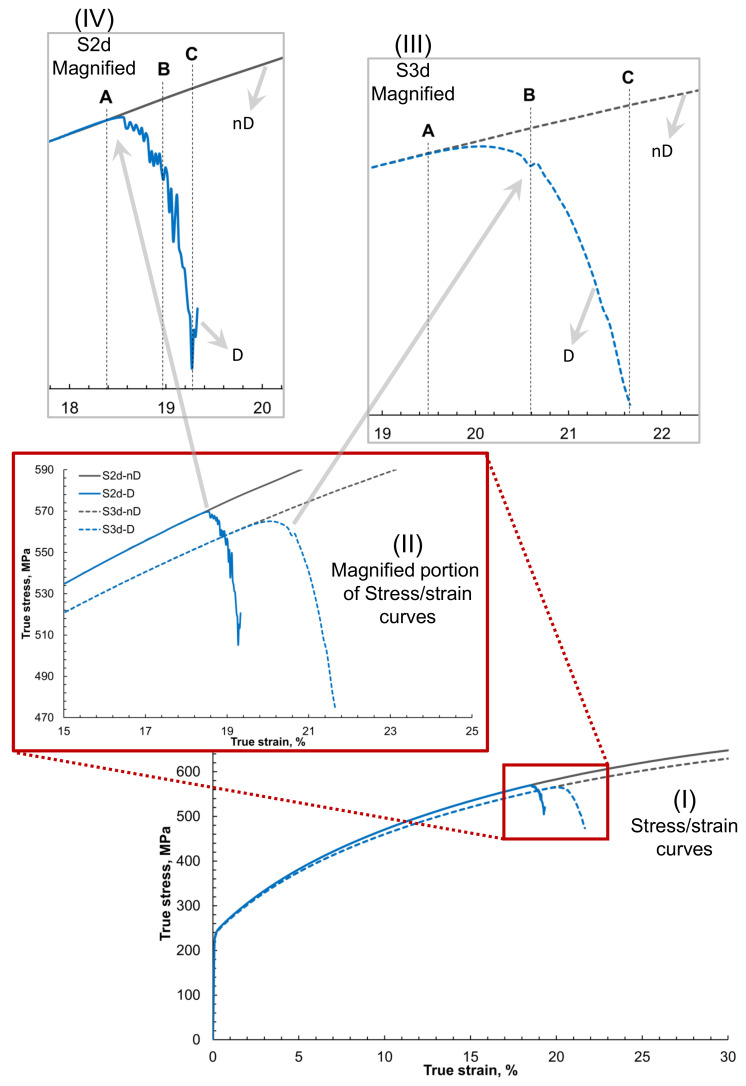
Global stress–strain curves of 2D and 3D RVE of steel with damage and without damage for comparison. Where (**I**) is showing complete curves up to 30% strain, (**II**) is the magnified area where the damage initiation occurs in the material, (**III**) is further magnified figure for S3d case, and (**IV**) magnified figure for S2d case. A, B, and C show the global strain values at which the local frames at damage initiation, evolution, and propagation are taken and shown.

In Figure 3 global stress–strain behavior of the 2D and 3D RVEs with damage and non-damage is presented. The figure is constructed such that starting from the overall stress–strain curve, the damage initiation, and evolution are focused for detailed understanding. In Figure 3I, it is observed that the stress–strain curves for 2D and 3D RVEs show the same trend and slope with 2D RVE slightly over-predicting the accumulated average von Mises true stress for a specific amount of strain.

As shown earlier in Figure 1, the 2D RVE has been constructed by stripping off all the layers in a 3D RVE except the top one. It is done to keep the same surface for quantitative and qualitative comparison of results from the same area (something possible in simulations but impossible experimentally). The ODF adopted to construct this virtual RVE is neutral for the bigger RVE but might be slightly biased to a particular direction for the first layer only. Therefore it yields slightly different results when loaded in monotonic tension. The ≤3% difference of the stress can be ignored in the current analysis. When damage is incorporated in the model, the matrix degradation around 20% of true strain is visible. This portion of the figure has been magnified and shown in Figure 3II. There is a threshold where the material in 2D and 3D steel gets computationally challenging, and the solution does not converge.

Although the same damage criteria for the ferrite matrix with critical plastic strain $\epsilon_{crit}$ being 0.5 was used, it is observed that the damage initiation in the case of 2D occurs at a global strain of 6.1% earlier in comparison with damage initiation in case of 3D. It probably occurs due to the higher contrast of accumulated strain in the case of 2D. The damage in the 3D steel case occurs at the later stage of global strain compared with 2D steel because of dislocation motion in the third direction and greater stress relaxation, as shown in Figure 3III,IV. This trend needs further exploration, which is carried out in the next section in more detail with the local stress and strain maps of respective RVEs.

It is observed that the damage propagation rate in the case of 2D is faster (Figure 3IV) and more abrupt. In 2D RVE, the damage evolution is convergent-divergent with sharp peaks of alternating valleys, whereas the stiffness degradation of the 3D RVE is smooth and convergent (Figure 3III). A, B and C frames were extracted during post-processing of the simulation results to compare local behaviors of the RVEs at the beginning, middle, and end of the damage, respectively.

#### 3.1.2. Local Behavior

**Figure 4:**
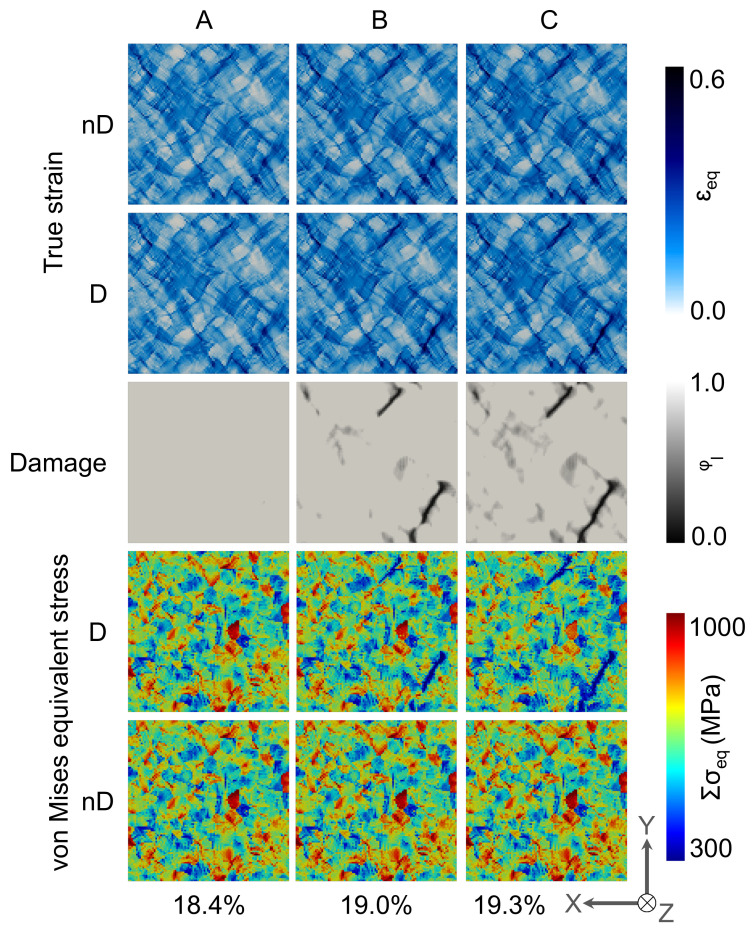
Local von Mises equivalent stress, local true strain, and damage evolution in 2D RVE at **A**, **B**, and **C** frames mentioned in Figure 3IV.

**Figure 5:**
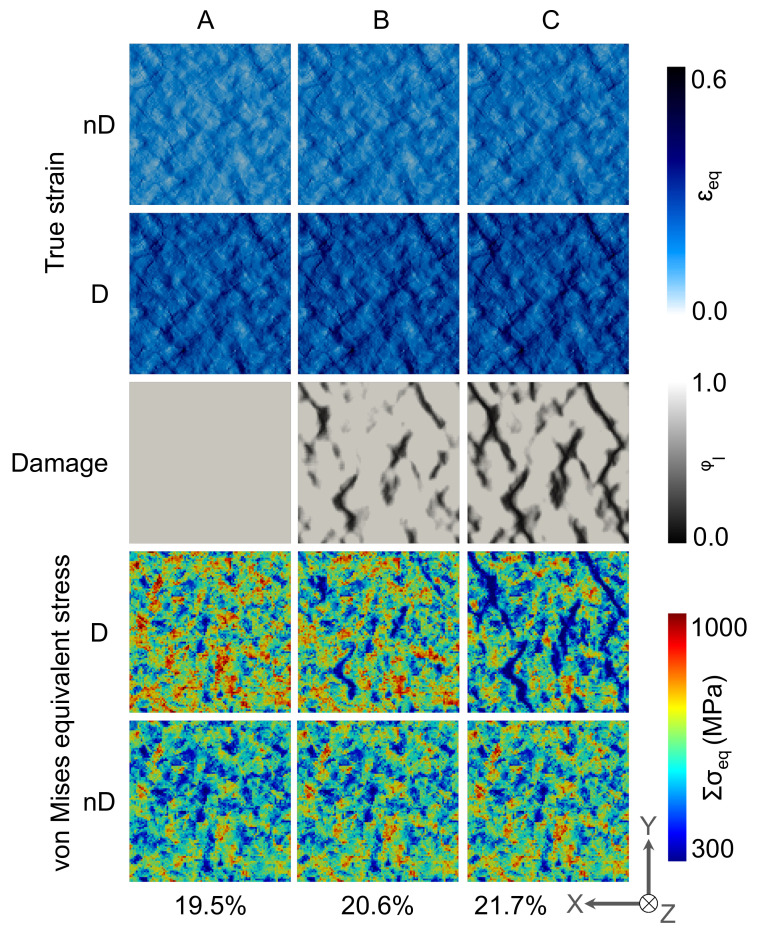
Local von Mises equivalent stress, local true strain and damage evolution in 3D RVE at **A**, **B** and **C** frames mentioned in Figure 3III.

The local strain, damage, and stress evolution at the A, B, and C frames on the top surfaces of the 2D and 3D RVEs are shown in Figure 4 and Figure 5, respectively. On a closer look at the strain distribution, it is observed that there are low strain plateaus in the grains not favorably aligned in the deformation direction. Between such plateaus, strain accumulation occurs in the form of narrow channels on the grain boundaries. This high contrast strain distribution further strengthens during further deformation (from A to C). These high contrast strain channels are aligned around $45^{{}^{\circ}}$ to the applied tensile load.

The damage initiation understandably occurs at the high strain zones in frame A. In frame B, the void coalescence occurs, and damage channels appear that propagate at $45^{{}^{\circ}}$ to the applied load as observed in frame C. Stress relaxation occurs near the damaged zones, which further assists in strain localization and damage propagation. Initially, the damage is inter-granular but starts growing trans-granularly with increased matrix degradation. As the 3D periodic boundary conditions are applied on the RVEs, this microstructure represents a columnar microstructure, and in such simulations, high stress and strain contrast is observed. 3D RVE simulations have been carried out to compare with the natural materials deformation behavior, and the results are presented in Figure 5.

In Figure 5, the strain distribution on the top surface of RVE in the case of 3D steel is shown for the non-damage and damage cases. In the case of the non-damage simulations, it is observed that the strain distribution in the steel matrix is more or less homogeneous, with few areas showing higher strain concentration zones. In the damaged RVE case, the strain contrast is higher as the subsurface damage influences the strain localization in specific regions. It results in high strain zones where the material damage propagates. It is observed that the damage propagation is $45^{{}^{\circ}}$ to the loading direction.

It is essential to mention here that the damage behavior shown in Figure 5 is only on the top surface. It is understood that there would be a complex damage network in 3D, which is challenging to show here. Looking at the top surfaces only and comparing the point to point data in C frames of damage in 2D RVE (Figure 4) and 3D RVE (Figure 5), the difference in the damage initiation sites and propagation zones is observed. Also, the stress accumulation on the surface in the 3D RVE case is lower, and the stress relaxation is higher as compared with the 2D RVE.

#### 3.1.3. Statistical Comparison of Local Stresses and Strains

**Figure 6:**
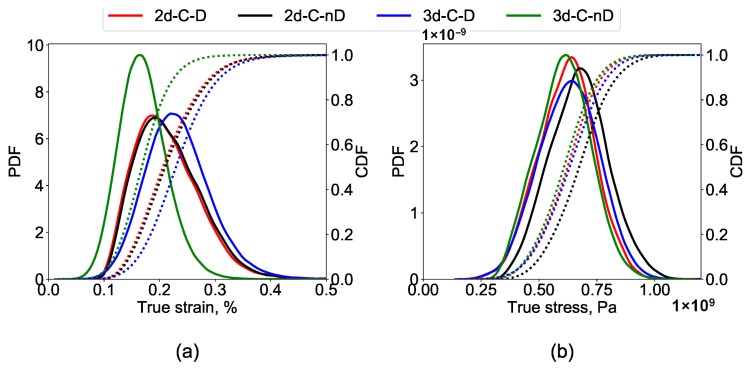
Probability and cumulative distribution in C frame of single-phase steel for (**a**) stress, and, (**b**) strain. The distribution is used to compare the behaviour of 2D and 3D RVEs in damages and non-damaged states.

A statistical analysis of the stress and strain distribution in the RVEs is carried out for an improved understanding of the before and after damage data. Such an analysis of the current simulation results is carried out by plotting probability distributions and cumulative distributions of the local stresses and strains for the C-frame (refer to Table 3 for details) of 2D and 3D RVEs. The results are shown in Figure 6. As shown earlier, the stress and strain distribution plots help understand the overall trends rather than just superficial data on top surfaces.

It is visible in Figure 6a that the peak strain in the 3d-C-nD is 40% lower than 3d-C-D, which reinforces the idea of the drastic difference of strain distribution in both cases due to damage progression as can be observed in Figure 5. The strain distribution is lower and localized in the 3D-nD case and is higher and distributed in the 3D-D case. Despite damage occurrence, there is no significant difference in the peaks and distribution for the case of 2d-C-D and 2d-C-nD (as shown in Figure 6a) which was also visually apparent in Figure 4. This occurs probably due to the 2D nature of the geometry, and the high strain localization zones merely change during damage evolution.

The comparison of local stress distributions is shown in Figure 6b. It is observed that in the case of 3D RVEs, the maximum stress magnitude remains consistent but is distributed in more elements after damage occurs. This enhanced stress distribution can be attributed to the 3D RVE nature. Whereas, in the case of 2D RVE, the stress distribution remains consistent, but the magnitude of the highest stress in the RVE drops by ≈12% after damage.

### 3.2. Deformation and Damage Behavior of DP-Steel

#### 3.2.1. Global Behavior

**Figure 7:**
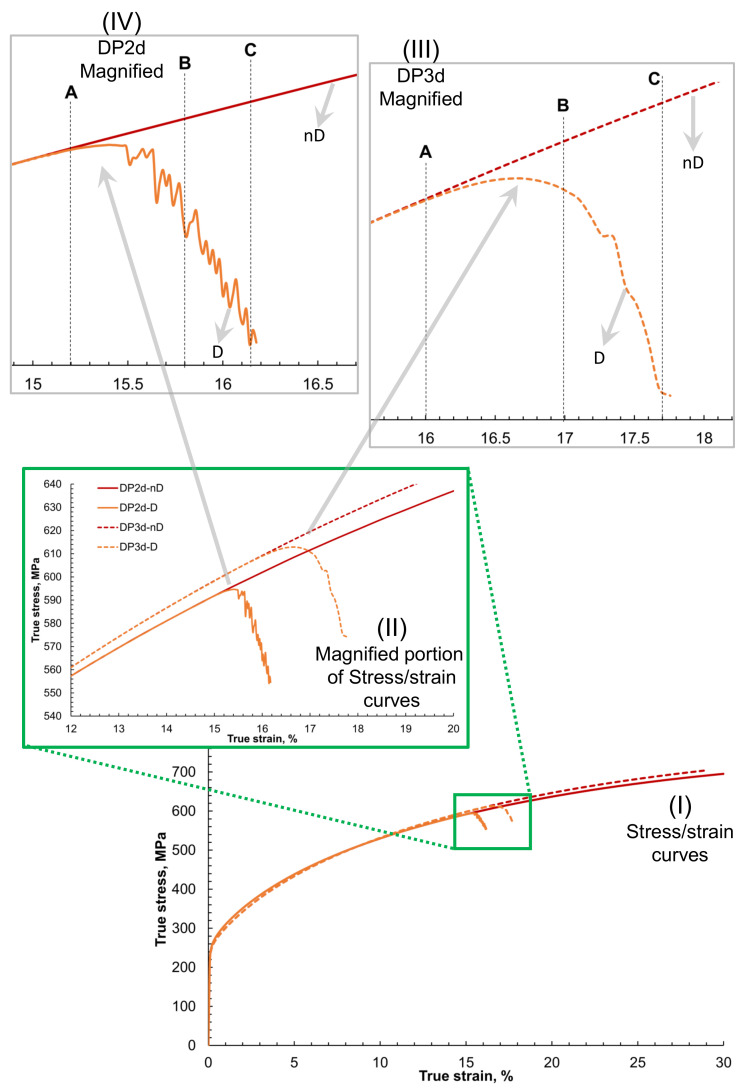
Global stress–strain curves of 2D and 3D RVE of DP-steel with damage and without damage for comparison. Where (**I**) is showing complete curves up to 30% strain, (**II**) is the magnified area where the damage initiation occurs in the material, (**III**) is a further magnified figure for DP3d case, and (**IV**) magnified figure for DP2d case. A, B, and C show the global strain values at which the local frames at damage initiation, evolution, and propagation are taken and shown.

The global stress–strain behavior of DP-steel is shown in Figure 7. It is observed that there is a slight difference in the global stress–strain behavior of 2D and 3D RVE, i.e., in Figure 7I. The flow stress at 30% of true strain in 3D RVE is 2% less than the 2D RVE case. This probably occurs due to a slight change in orientation distributions and percentage martensite on the surface. The overall flow curve for both cases is the same, and hence for point-to-point local comparison, these differences are ignored. Defining the damage criteria for the ferrite phase induces damage in the 2D RVE at 15% of strain, which is 6.3% less than the strain at which damage is induced in 3D RVE. This trend is similar to what is observed in the single-phase steel case.

It is observed that for the case of 3D RVE, the damage propagation is smoother and slower, as compared to the case of 2D RVE, where the damage propagation was non-uniform, and the degradation slope was higher. To compare the local strain, stress, and damage behavior at different stages with the non-damaged RVEs, 3 frames A, B, and C at the beginning, in the middle, and at the end of the damage are taken.

#### 3.2.2. Local Behavior

**Figure 8:**
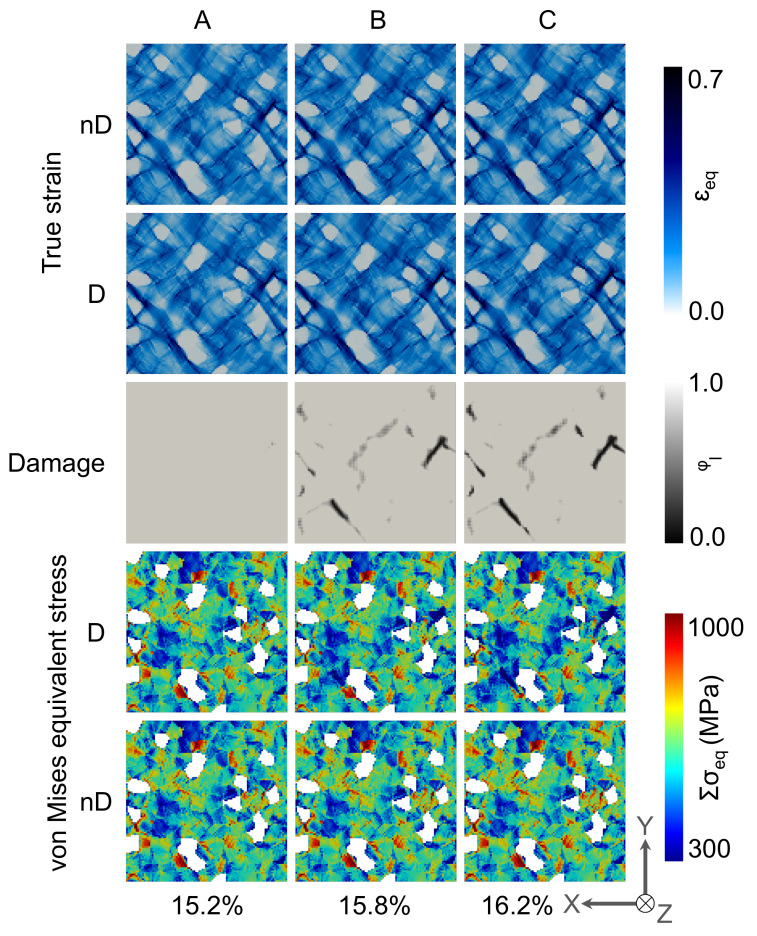
Local von Mises equivalent stress, local true strain and damage evolution in 2D RVE at **A**, **B** and **C** frames which are mentioned in Figure 7IV. High stress accumulations (~3 GPa) in the martensite grains distort the scale and therefore, martensite phase is not shown in the stress distribution maps.

In Figure 8 the deformation behaviour of the 2D RVE for the DP-steel case is shown for non-Damaged(-nD-) and damaged(-D-) cases. Comparing the local stress and strain distribution in Figure 8, it is observed that there is no significant difference in the case of stress and strain evolution in the damage and non-damaged case. The strain localization occurs in the vicinity of the martensite grains at $45^{{}^{\circ}}$ to the applied loading direction, whereas most of the ferrite matrix undergoes moderate strain change. In these high strain zones, the damage initiates and propagates due to which—in certain zones—stress relaxation occurs.

**Figure 9:**
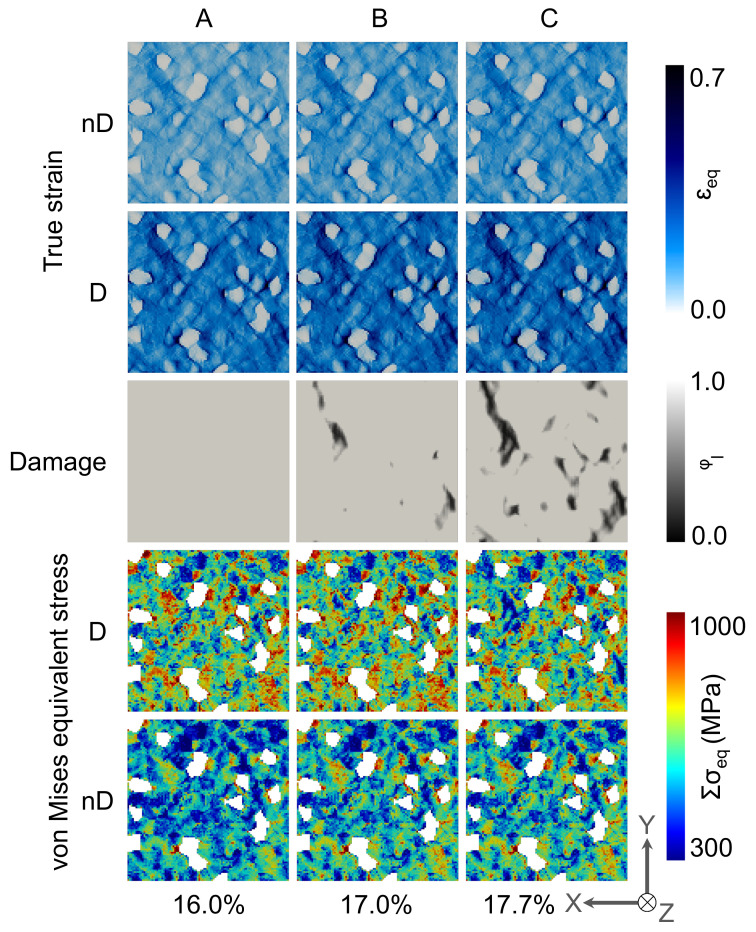
Local von Mises equivalent stress, local true strain and damage evolution in 3D RVE at **A**, **B** and **C** frames which are mentioned in Figure 7III. High stress accumulations (~3 GPa) in the martensite grains distort the scale and therefore, martensite phase is not shown in the stress distribution maps.

For the case of 3D RVE, i.e., the results are shown in Figure 9, it is observed that the strain is more homogeneously distributed in the ferrite matrix with high strain accumulation around the edges of martensite grains and between the closely packed martensite grains. In the case of damage incorporation, relatively higher strain concentrations are observed on the top surface of the RVE. It is observed that the damage does initiate and propagate in the vicinity of the martensite grains. Still, it branches through the whole matrix, resulting in relatively slower degradation, as seen in Figure 7III. Around a few martensite grains, damage does not initiate at all. The crack branching can be observed for 3D RVE simulations as the crack has 3rd dimension to grow into, which affects the damage evolution drastically compared with damage evolution in 2D RVE.

The damage initiation and propagation behaviour of 2D-RVE (Figure 8) and 3D-RVE (Figure 9) is compared. From these results, it can be stated that the damage initiation can be nicely predicted by using 2D RVEs, but 3D RVE simulations more accurately capture damage propagation. Due to damage propagation, stiffness degradation of the matrix occurs, the stress shifts to the non-damaged matrix, and, therefore, some high-stress concentration zones are observed. The stress concentration and strain localization are usually higher on the Martensite/Ferrite interface, but this is not always the case. The data at C-frames is statistically analyzed for a better understanding of the distributions of mechanical attributes.

#### 3.2.3. Statistical Comparison of Local Stresses and Strains

**Figure 10:**
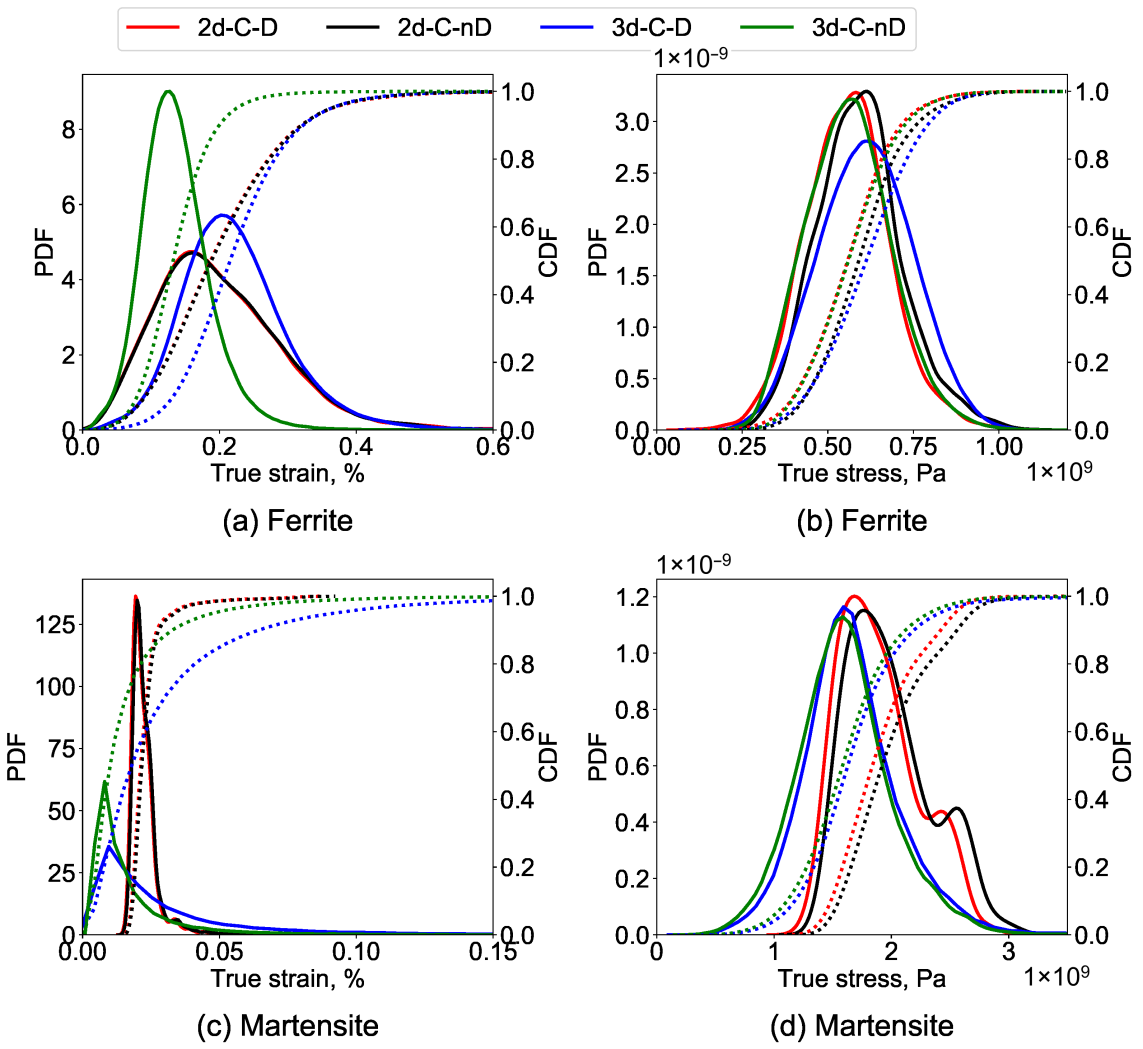
Probability and cumulative distribution in C frame of DP-steel case, (**a**) strain distribution in ferrite matrix, (**b**) stress distribution in ferrite matrix, (**c**) strain distribution in martensite grains and (**d**) stress distribution in martensite grains, is compared. The distributions are compared to observe the overall stress and strain behaviour of 2D and 3D RVEs in damaged and non-damaged states.

For a detailed comparison of local stress and strain distributions, the last frames of the DP-steel simulations were processed (refer to the provided data in Table 3) to obtain PDFs and CDFs for each phase using the statistical analysis. The results for the strain and stress distribution in the ferrite phase are provided in Figure 10a,b, respectively. The strain and stress distribution in the martensite phase are provided in Figure 10c,d, respectively.

It is observed in Figure 10a that for the ferrite phase, the peak of strain distribution in 3D-D is ≈45% higher than in the case of 3D-nD due to damage progression. Whereas, in the case of 2D, similar peaks and similar strain distribution are observed even after damage. The strain distribution in the hard martensite phase is shown in Figure 10c. It is observed that the strain in the martensite phase is very low and more localized. The strain in martensite grains is ≈60% higher in 2D simulations than in 3D simulations.

The comparison of the stress distribution for the ferrite phase is shown in Figure 10b. It is observed that there is no significant difference in magnitude and distribution of the 2D cases, whereas, in the case of 3D RVEs, the maximum stress magnitude remains consistent but is distributed in more elements after damage occurs. It is also interesting to note that the stress and strain distribution magnitudes and trends before and after damage are similar when comparing (a) and (b) in Figure 6 and Figure 10. The inclusion of the second phase particles in the case of DP-steel makes all the difference in the observed patterns.

For the martensite phase, i.e., in Figure 10d, the stress distribution for the 3D cases is similar to the maximum stress of around 1.5 GPa. For the 2D cases, the bi-modal stress distribution is observed with a slight decrease in the maximum stresses after damage in the ferrite matrix due to stress relaxation.

## 4. Discussion

In the current work, a crystal plasticity-based numerical simulation model was developed to analyze the local stress and strain evolution in single-phase steel and multi-phase DP-Steel. The RVEs were constructed using Dream.3D by adopting microstructural attributes from the previous study of Qayyum et al. [23]. The virtually constructed micro-structures, as shown in Figure 1 and Figure 2, majorly consist of ellipsoid grains without any sharp edges or narrow artifacts.

In Figure 3 and Figure 7, it is observed that during plastic deformation, the global stress–strain in 2D and 3D cases are similar. With the introduction of damage criteria for the ferrite phase, damage initiates and propagates through the matrix, causing stiffness degradation. This trend is similar to what has been reported earlier by researchers using similar material models [5,26,27]. Experimental testing was not in the scope of the current work. Therefore, the accuracy of the obtained results can be analyzed by comparing trends with the previously published work [13,38]. The currently obtained simulation results seem reasonably close to the already reported trends in the literature [39].

**Figure 11:**
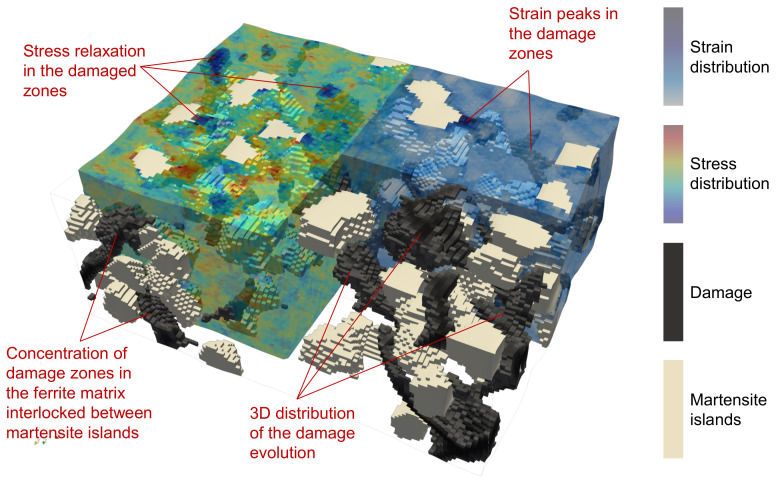
Schematics showing distribution of the damage in the 3D RVE with semi transparent stress and strain distribution in some areas. The stress and strain distribution in the ferrite phase is shown. Martensite phase is shown in the white colour. Damage is shown in the black colour. Different phenomena occurring during damage evolution are labelled in red.

A relatively simple ductile damage criterion was incorporated in the numerical simulation model for the ferrite phase. For simplicity, it was assumed that the brittle cracking of martensite does not occur in the applied loading regime, and therefore, was neglected. It is observed that the local strain heterogeneity increases with the increasing global strain in ferrite-steels and DP-Steels. Intense localization of shear strain in ferrite grains occurs at higher global strains, which results in damage initiation. In ferrite-steels, the damage zones are usually the triple points or the grain boundaries of high contrast grains. In DP-Steels, the damage zones are the high strain contrast ferrite/martensite grain boundaries, which provide an easy pathway for damage evolution when oriented along the direction of maximum resolved shear stress in the material. The inter-dependent phenomena co-occurring during deformation are presented schematically for the case of DP-Steel in Figure 11.

It is observed that the damage initiation in the case of 2D-RVE occurs at a global strain of ≈6% earlier in comparison with damage initiation in the case of 3D-RVE. This trend is exciting and probably occurs due to the higher contrast of accumulated strain in the case of 2D, as reported by earlier researchers [17,23]. These observations help generalize that damage initiation in 2D-RVE occurs at ≈6% less global strain than the damage initiation in 3D-RVE. This can imply one of the following outcomes:

1. Either it could be taken and understood as it is and be informed that in 2D RVEs, the damage initiates at ≈6% less strain. Or,
2. It can be used to apply a correction factor for increasing the damage criteria by ≈6% for 2D RVE case to match the results with 3D RVE results.

Initially, the damage is inter-granular but starts growing trans-granular with increased damage in the matrix. This is how the actual damage initiation and propagation due to applied tensile load have been reported earlier [26]. Due to the restricted flow of stresses and strain in the 3rd direction, the evolution of damage networks in 2D is different from in 3D.

For multi-dimensional data sets, statistical data analysis for the comparison of local attributes for the CP based full phase simulations have been employed by several researchers in the past [5,17,21,40] to get a better understanding of the local distribution of the attributes. In the current work, the probability and cumulative distributions of the stress and strains for each phase in the last frames of the 2D and 3D-RVEs are compared. The stress and strain distributions in 2D-RVEs remain unchanged in the ferrite phase even after adequate damage evolution has occurred. In the case of 3D-RVE, it is observed that the damage evolution results in higher strain and higher stress localization, which is close to experimental observations [24,28].

This study provides valuable information for understanding the limitations of RVEs chosen for full-phase crystal plasticity simulations. It provides insight into the different damage initiation sites and propagation speed for 2D or 3D RVE. Such information is vital for confidently moving forward with the damage simulations. The current research will help move towards a conclusive approach with a comprehensive model that will satisfy the experimental observations and accurately yield the local deformation behaviors during deformation and damage under specified loading conditions. Further comparison with experimental data in the future will help in understanding the accuracy of the results.

## 5. Conclusions

Selection of an appropriate RVE for mesoscale simulations is a challenging task. In the current work, CP-based full-phase simulations are run, and a comparison of global and local stress, strain, and damage evolution in 2D and 3D RVEs is performed systematically. The obtained results provide an insight into the interplay of the mechanical responses and how they are affected by the dimensional differences in the considered RVEs. The study can be concluded as follows:

1. Numerical simulation modeling approach of damage initiation and propagation in single-phase steel and DP-steel for 2D, and 3D RVEs is successfully developed.
2. The damage in case of 2D RVE initiates earlier than in 3D RVE. This should be kept in mind for implementing full phase CP-simulations results to design problems.
3. The statistical analysis of the simulation results in the form of PDFs and CDFs for each phase proves to be a viable tool for comparative analysis of the overall mechanical response. The stress and strain distributions in the ferrite phase are similar for single and multi-phase materials. The enhanced mechanical properties of the DP-steel are an outcome of the incorporated hard martensite phase, which undergoes true strains close to 0.02% and stresses as high as 1.5 GPa.
4. Similar to what has been reported in the literature before, it is observed that the local damage initiation and evolution in single-phase steel is at triple points or grain boundaries which propagates $45^{{}^{\circ}}$ to the deformation direction. The local damage in DP-steel is at the ferrite-martensite grain boundaries or in the ferrite grains between the martensite islands and propagates in a complex network.
5. 2D RVEs can be adopted to reasonably predict where and when the damage will initiate in the matrix, but the 3D RVEs should be adopted to predict the consistent and reliable results regarding the evolution of the damage network matrix degradation. Experimental validation of these results is needed to be carried out in future.

## Figures and Tables

**Table 1:** Physical and fitting parameter values for ferrite and martensite used for the numerical simulation model definition, Adopted from the earlier published literature [36].

Parameter Definition	Symbols	Attributes for Ferrite	Attributes for Martensite	Unit
First elastic stiffness constant with normal strain	$C_{11}$	233.3	417.4	GPa
Second elastic stiffness constant with normal strain	$C_{12}$	135.5	242.4	GPa
First elastic stiffness constant with shear strain	$C_{44}$	128.0	211.1	GPa
Shear Strain rate	${\dot{\gamma }}_{0}$	1	1	$10^{-3}$/s
Initial Shear resistance on [111]	$S_{0}\left[111\right]$	95	406	MPa
Saturation shear resistance on [111]	$S_{\infty }\left[111\right]$	222	873	MPa
Initial Shear resistance on [112]	$S_{0}\left[112\right]$	96	457	MPa
Saturation shear resistance on [112]	$S_{\infty }\left[112\right]$	412	971	MPa
Slip hardening parameter	$h_{0}$	1000	563	MPa
Interaction hardening parameter	$h_{\alpha ,\beta }$	1.0	1.0	-
Stress exponent	*n*	20	20	-
Curve fitting parameter	*w*	2.0	2.0	-

**Table 2:** Physical and fitting damage parameter values used for ferrite phase, adopted from literature [16].

Parameter Definition	Symbols	Value	Unit
Interface energy	$g_{0}$	1.0	J m^−2^
Characteristic length	$l_{0}$	1.5	μm
Damage mobility	*M*	0.01	s^−1^
Damage diffusion	*D*	1.0	-
Critical plastic strain	$\epsilon_{crit}$	0.5	-
Damage rate sensitivity	*P*	10	-

**Table 3:** Nomenclature adopted in the current work for the representation of simulation results.

Material	RVE Type	Damage Inclusion	Point	Increment	Nomenclature
DP-steel	2D	D	A	1203	DP2d-A-D
			B	1386	DP2d-B-D
			C	1437	DP2d-C-D
		nD	A	1900	DP2d-A-nD
			B	2000	DP2d-B-nD
			C	2050	DP2d-C-nD
	3D	D	A	1310	DP-3d-A-D
			B	1520	DP3d-B-D
			C	1600	DP3d-C-D
		nD	A	1300	DP3d-A-nD
			B	1550	DP3d-B-nD
			C	1600	DP3d-C-nD
Steel	2D	D	A	1600	S2d-A-D
			B	1750	S2d-B-D
			C	1770	S2d-C-D
		nD	A	2400	S2d-A-nD
			B	2550	S2d-B-nD
			C	2600	S2d-C-nD
	3D	D	A	1770	S-3d-A-D
			B	1990	S3d-B-D
			C	2080	S3d-C-D
		nD	A	1800	S3d-A-nD
			B	2000	S3d-B-nD
			C	2100	S3d-C-nD

**Figure A1:**
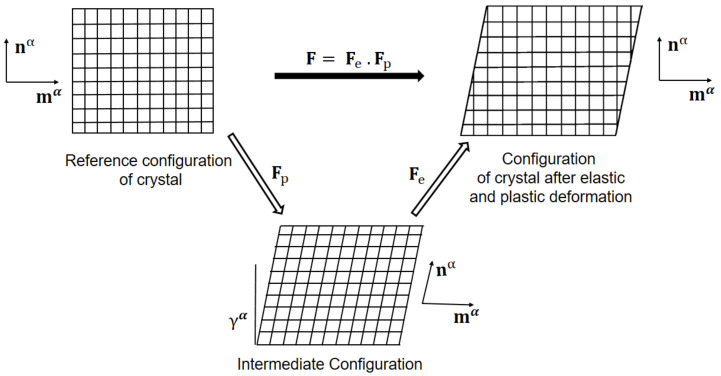
Varying configurations of the crystal during deformation from reference to elastic to plastic deformation modes.

## Data Availability

The simulation data is not publicly available but can be shared upon request.

## Abbreviations

RVE	Representative Volume Element	–
EBSD	Electron Back Scatter Diffraction	–
CP	Crystal Plasticity	–
TRIP	Transformation Induced Plasticity	–
ESD	Estimated Sphere Diameter	–
Mg-PSZ	Magnesium Partially Stabilized Zirconia	–
PDF	Probability Distribution Function	–
CDF	Cumulative Distribution Function	–
ODF	Orientation Distribution Function	–
-D-	Damage Case	–
-nD-	Non-Damage Case	–
IPF	Inverse Pole Figure	–
2D	Two Dimensional (geometry)	–
3D	Three Dimensional (geometry)	–
FFT	Fast Fourier Transformation	–
$G_{N}$	Number of considered grains	–
$D_{f}$	Mean feature estimated sphere diameter	m
$g_{0}$	Interface energy	Jm${}^{-2}$
$l_{0}$	Characteristic length	m
${\dot{F}}_{ij}$	Macroscopic rate of the deformation gradient	s${}_{-1}$
$P_{ij}$	Macroscopic first Piola–Kirchhoff stress tensor	Pa
*M*	Damage mobility	s${}^{-1}$
$\epsilon_{crit}$	Critical plastic strain	–

## Appendix A. Phenomenological Crystal Plasticity Model

The total deformation gradient F in crystal plasticity formulation written in Equation (A1) comprises of the product of elastic and plastic deformation gradients, which are shown in the Figure A1.

(A1) $$F=F_{e}\;\cdot \;F_{p}$$

The First Piola-Kirchoff stress P is given in Equation (A2) as measured from Cauchy stress tensor σ and elastic deformation gradient $F_{e}$.

(A2) $$P=\left(det\;\cdot \;F_{e}\right)\sigma \;\cdot \;F_{e}^{-t}$$

where Cauchy Stress (or true local stress) tensor is measured from the force acting on the element’s deformed configuration, the first Piola-Kirchoff stress P is the unsymmetric tensor because it has one of its index attached to the material reference configuration of the element while another index is in the deformed configuration of the element. Therefore, second Piola-Kirchoff stress S is used as a symmetric tensor which is the work conjugate of Green-Lagrangian strain tensor E and it is defined as

(A3) $$S=C\;\cdot \;E_{e}=F_{e}^{-1}\;\cdot \;P$$

The C in above expression is called the elastic stiffness tensor. In crystalline materials the plastic deformation gradient $F_{p}$ evolves into Plastic velocity gradient $L_{p}$ caused by dislocation slip. The latter further depends on the Schmidt’s factor $(m^{\alpha }$⊗$n^{\alpha })$ which is stated as:

(A4) $${\dot{F}}_{p}=L_{p}\;\cdot \;F_{p}$$

(A5) $$L_{p}=\dot{\gamma^{\alpha }}\left(m^{\alpha }⊗n^{\alpha }\right)$$

Shear strain rate $\dot{\gamma^{\alpha }}$ on the α slip system depends upon the reference shear rate $\dot{\gamma_{0}}$, initial stress on α slip system $S^{\alpha }$, and the exponent *n* is stated in Table 1. The $S^{\alpha }$ and dependence matrix $q_{\alpha \beta }$ express the localized strain hardening rule for ferrite phase [32].

The values of α can vary depending upon the number of slip systems available in the material α = 1, 2, 3…, $N_{slip}$

(A6) $$\dot{\gamma^{\alpha }}=\dot{\gamma_{0}}{\left|\frac{\tau^{\alpha }}{S^{\alpha }}\right|}^{n}sgn\left(\tau^{\alpha }\right)$$

(A7) $$\dot{S^{\alpha }}=h_{0}{\left(1-\frac{S^{\alpha }}{S_{s}}\right)}^{w}q_{\alpha \beta }\left|\dot{\gamma^{\beta }}\right|$$
